# Œnothera Biennis in Mucous Inflammations

**Published:** 1878-02-20

**Authors:** James F. Sullivan


### ŒNOTHERA BIENNIS IN MUCOUS INFLAMMATIONS.

By James F. Sullivan, M.D.

Œnothera Biennis, or Evening Primrose, which is indigenous to nearly all parts of the United States, possesses valuable properties as a mild sedative and alterative in many diseased conditions of mucons surfaces, especially the gastric, intestinal and vesical. It is useful in some forms of dyspepsia, particularly those accompanied by an irritable state of the stomach and of the bladder, as indicated by frequent vomiting and micturition. Having prescribed the remedy for eight years, I have been able to carefully note its effects, and am convinced it will be an important addition to our list of medicines. A brief history of a few cases will best illustrate its action.

Case 1, A man, aged 26, of active habits, had dyspepsia for five years. His most distressing symptom was an almost constant pain in the region of the bladder, with frequent micturition. He had been treated in various ways without benefit, and was more than once sounded for stone. He was given half a drachm of fluid extract of œnothera with a drachm of tincture cinchona comp. in water, just before meals, with immediate and permanent relief.

ase 2. A gentleman, who had been a dyspeptic for many years, had suffered especially from frequent vomiting of food, distress after eating, and restlessness at night, which was aggravated by a desire to urinate. Half a drachm of the fluid extract, just before eating and at bedtime, promptly checked the vomiting, allayed the irritability of the bladder, and gave him refreshing rest at night.

Case 3. Mr. M., aged 40, while convalescing from typhoid fever, was attacked with an obstinate dysentery with severe tenesmus and frequent discharges or bloody mucus. For five days the disease resisted every known remedy, including anodyne enemas, calomel and opium, turpentine emulsion, etc. All other treatment was then discontinued, and twenty-five drops of fluid extract of œnothera was given every three hours. The dysenteric discharges entirely ceased after the second dose, and the patient had a natural fecal discharge within twelve hours.

Dr. N. S. Davis of Chicago, has recently found valuable results from this remedy (*Quarterly Abstract of Medical Science*, February, 1877). He says: "From my own clinical observation I am inclined to regard it as a mild but efficient sedative to nervous sensibility, acting more especially on the pneumogastric nerve."

Its chief value, I believe, will be found in typhoid fever, to the treatment of which it is peculiarly adapted by its soothing action upon the intestinal mucous surface. I am convinced that it essentially modifies the inflammatory condition which precedes ulceration of Peyer's patches, and that its use may frequently prevent ulceration. The usual dose in typhoid fevers is from fifteen to thirty drops every three hours. There is no danger of an overdose, and I have known a tablespoonful of the fluid extract to be given every two hours, by mistake, till two ounces had been taken. In that case it seemed to revive the patient, after the manner of a stimulant, and I am not sure that it might not be advantageously given in large doses in some cases. The fluid extract of œnothera is not incompatible in mixtures with any other medicine. Its flavor resembles that of cold tea, and it is acceptable to any condition of the stomach. It appears to be well worthy the attention of the profession, and the writer would be pleased to learn the results of its use in the hands of other practitioners.—*Pacific Medical Journal.*

